# Paramyxovirus Diversity within One Population of *Miniopterus fuliginosus* Bats in Sri Lanka

**DOI:** 10.3390/pathogens11040434

**Published:** 2022-04-02

**Authors:** Therese Muzeniek, Thejanee Perera, Sahan Siriwardana, Fatimanur Bayram, Dilara Bas, Mizgin Öruc, Beate Becker-Ziaja, Inoka Perera, Jagathpriya Weerasena, Shiroma Handunnetti, Franziska Schwarz, Gayani Premawansa, Sunil Premawansa, Wipula Yapa, Andreas Nitsche, Claudia Kohl

**Affiliations:** 1 Centre for Biological Threats and Special Pathogens, Highly Pathogenic Viruses (ZBS 1), Robert Koch Institute, 13353 Berlin, Germany; muzeniekt@rki.de (T.M.); kaplanf@rki.de (F.B.); basd@rki.de (D.B.); oerucm@rki.de (M.Ö.); schwarzf@rki.de (F.S.); nitschea@rki.de (A.N.); 2 Institute of Biochemistry, Molecular Biology and Biotechnology, University of Colombo, Colombo 00300, Sri Lanka; thejanee90@gmail.com (T.P.); jagath@ibmbb.cmb.ac.lk (J.W.); shiromah@ibmbb.cmb.ac.lk (S.H.); 3 Identification of Emerging Agents (IDEA) Laboratory, Department of Zoology and Environment Sciences, University of Colombo, Colombo 00300, Sri Lanka; sahan@zoology.cmb.ac.lk (S.S.); icperera@sci.cmb.ac.lk (I.P.); suviprema@gmail.com (S.P.); wipula@gmail.com (W.Y.); 4 Centre for International Health Protection, Public Health Laboratory Support (ZIG 4), Robert Koch Institute, 13353 Berlin, Germany; beate.becker-ziaja@bnitm.de; 5 Colombo North Teaching Hospital, Ragama 11010, Sri Lanka; gavisprema@gmail.com

**Keywords:** bat Paramyxovirus, *Miniopterus fuliginosus*, Sri Lanka

## Abstract

Bats are known as typical reservoirs for a number of viruses, including viruses of the family Paramyxoviridae. Representatives of the subfamily Orthoparamyxovirinae are distributed worldwide and can cause mild to fatal diseases when infecting humans. The research on Paramyxoviruses (PMVs) from different bat hosts all over the world aims to understand the diversity, evolution and distribution of these viruses and to assess their zoonotic potential. A high number of yet unclassified PMVs from bats are recorded. In our study, we investigated bat species from the families Rhinolophidae, Hipposiderae, Pteropodidae and Miniopteridae that are roosting sympatrically in the Wavul Galge cave (Koslanda, Sri Lanka). The sampling at three time points (March and July 2018; January 2019) and screening for PMVs with a generic PCR show the presence of different novel PMVs in 10 urine samples collected from *Miniopterus fuliginosus*. Sequence analysis revealed a high similarity of the novel strains among each other and to other unclassified PMVs collected from *Miniopterus* bats. In this study, we present the first detection of PMVs in Sri Lanka and the presence of PMVs in the bat species *M. fuliginosus* for the first time.

## 1. Introduction

The virus family Paramyxoviridae (PMV) belongs to the order Mononegavirales and represents large, enveloped viruses with a negative-sense ssRNA genome [1]. The family is further divided into several subfamilies, from which the Orthoparamyxovirinae is divided into eight genera. Representative viruses of these genera are able to infect a broad range of hosts, including reptiles, birds, fish and mammals [2]. Within the Orthoparamyxovirinae, human pathogenic viruses are described in the genera Respirovirus (e.g., human respirovirus 1 and 3), Morbillivirus (e.g., Measles virus) and Henipavirus (e.g., Hendra virus, Nipah virus) [3]. Bats within the order Chiroptera are known to host a high variety of viruses, including zoonotic viruses. Their ability to carry the viruses without suffering from viral infections makes them a research topic of high interest with respect to immune response [4]. It is assumed that this is a result of a long-term co-evolution of viruses and bats [5]. Unique features of these mammals, such as their ability to fly, their migration patterns over long distances and their roosting habits in large colonies, promote them as suitable reservoirs for viruses [6]. Due to the intensified research on bat pathogens, the number of identified yet unclassified bat paramyxoviruses rose in the past decades [3]. Some zoonotic PMVs have a worldwide distribution; some highly pathogenic PMVs such as Nipah virus, Hendra virus and Menangle virus are recorded in Asia and Australia only [7]. Therefore, a regular screening and monitoring of viruses in bats and the characterization of novel PMVs can help to understand the evolution, distribution, zoonotic potential and transmission routes of these viruses. 

With 31 recorded species, Chiroptera is a highly represented order of mammals in Sri Lanka [8]. Although bat species, their habitats, distribution and ecology are common research fields for zoologists in Sri Lanka, there are only a few studies available about their role as reservoirs for zoonotic viruses [9,10,11,12]. In our study, we examined samples from different bat species of the families Rhinolophidae, Hipposiderae, Pteropodidae and Miniopteridae, roosting together in one of the largest natural caves in Sri Lanka (Wavul Galge cave, Koslanda). In previous investigations, we reported alpha- and beta-coronaviruses in rectal swabs and feces samples of the two species *Miniopterus fuliginosus* and *Rousettus leschenaultii* [9]. Since all of the bat families inhabiting the cave were also known to carry PMVs, we aimed to screen all species for novel PMVs and concluded the zoonotic risk originating from the Wavul Galge cave. With this article, we present the molecular detection of different bat paramyxovirus strains for the first time in a population of *M. fuliginosus* bats and for the first time in Sri Lanka.

## 2. Results

In our study, we investigated a total of 143 urine samples that were collected at three sampling points in March and July 2018 and January 2019. An overview of the collected urine samples per sampling point and bat genus is shown in Table 1. Samples were obtained from the genera Hipposideros, Rhinolophus, Rousettus and Miniopterus. A majority of 102 urine samples from Miniopterus bats were collected during the sampling session in July 2018 and the remaining 11 were collected in January 2019. All collected samples were screened for paramyxoviruses using the semi-nested PCR assay and 10 urine samples from *M. fuliginosus* bats collected in July 2018 tested positive. De novo assembly of the Sanger sequences revealed the presence of three different PMV strains in the samples, sharing nucleotide (nt) identities between 80 and 81%.

**Figure 1:**
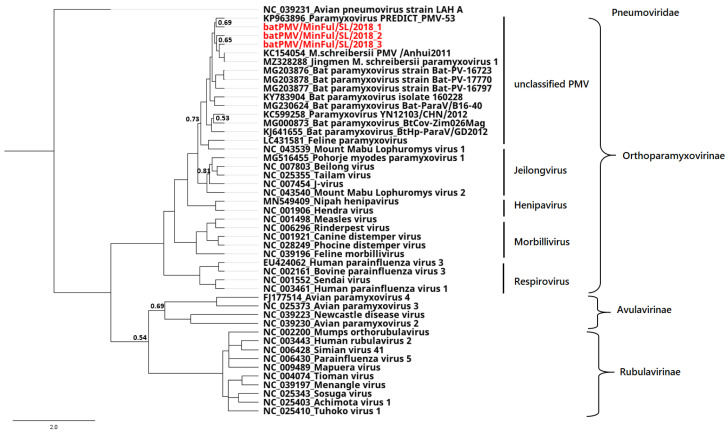
Phylogenetic tree based on a 473 nt alignment, sequence is located on the L Gene. The novel strains batPMV/MinFul/SL/2018_1–3 (red), selected PMV strains and representative Avulavirinae and Rubulavirinae were used for phylogenetic reconstruction. The avian pneumovirus strain LAH A (NC_039231) was included as an outgroup for the calculation. The phylogenetic tree was calculated with the Bayesian algorithm, and 1 million generations were calculated with a sub-sampling frequency of 100 and a burn-in of 10%. Substitution model GTR was selected with a gamma-distributed rate variation. Posterior probability values of <0.9 are shown next to the respective nodes.

In the phylogenetic reconstruction (Figure 1), the three novel strains cluster monophyletically with a number of unclassified PMVs. Representatives of the genus Jeilongvirus build a paraphyletic branch close to the unclassified PMVs. The genera Henipavirus, Morbillivirus and Respirovirus appear as separate branches inside the clade of the Orthoparamyxovirinae subfamily. The selected representatives of Rubulavirinae and Avulavirinae subfamilies were reconstructed as clearly distinct clades.

**Figure 2:**
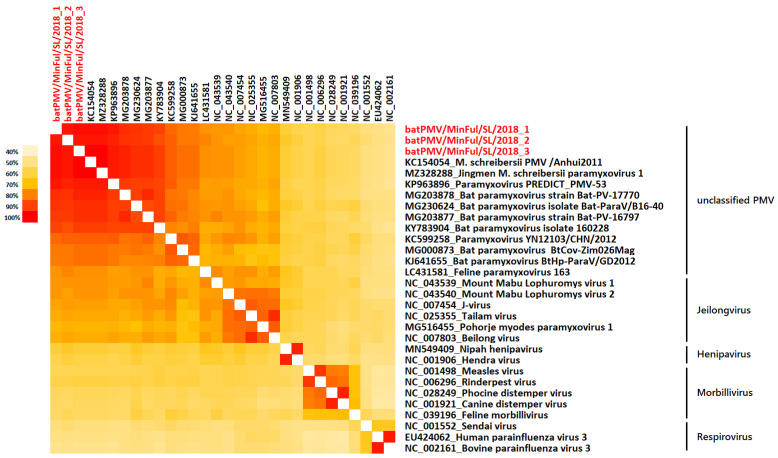
Heatmap based on a 156 aa alignment of the L gene coding for the RNA polymerase protein. The novel strains batPMV/MinFul/SL/2018_1–3 (red) and selected PMV strains belonging to the subfamily Orthoparamyxovirinae were used for alignment and heatmap calculation.

The phylogenetic results are confirmed by a heatmap based on a multiple aa sequence alignment, including selected representative strains (Figure 2). 

The Orthoparamyxovirinae share identities between 45.5% and 100% among each other. In general, high identities of the novel strains batPMV/MinFul/SL/2018_1–3 to other unclassified PMVs were calculated, ranging between 76.9 to 100%. The three novel strains share identities of 98.1% on aa level. High similarities were calculated to other bat PMVs and all novel strains share identities between 91 and 100% to other *Miniopterus* PMVs. The strain batPMV/MinFul/SL/2018_3 shares a 100% identity with two unclassified PMV strains (KC154054, MZ328288), which were both collected from *Miniopterus schreibersii* in China. In contrast, the novel strain batPMV/MinFul/SL/2018_2 shares a slightly lower identity of 96.8% to other strains from Sri Lanka. 

The aa identities of the three novel strains to representatives from the genus Jeilongvirus are comparably high (71.2–76.9%). In contrast, they share lower identities to Henipaviruses (59–64.1%), Respiroviruses (55.1–60.3%) and Morbilliviruses (49.4–53.8%).

## 3. Discussion

These results demonstrate the first detection of PMVs in Sri Lankan bats and further the first detection within the species *M. fuliginosus* worldwide. In general, PMVs are known to be present on all populated continents [2,13]. Although PMVs can be found in a number of hosts such as reptiles, birds, fish and other mammals, only chiroptera-hosted PMVs are known to cause zoonotic diseases in humans [14].

Pteropodidae are natural hosts for henipaviruses, causing respiratory and neurological diseases in humans with high cases of fatalities [15]. On one hand, other PMVs of the genera Rubulavirus (mumps virus), Respirovirus (human respirovirus) and Morbillivirus (measles virus) are known to cause zoonotic diseases highly pathogenic to humans worldwide [14]. On the other hand, there is little knowledge about the increasing number of unclassified PMVs. A high number of these identified strains were detected in bats of the family Miniopteridae. In our phylogenetic reconstruction of the three novel PMV strains from Sri Lanka, batPMV/MinFul/SL/2018_1–3 were assigned to the same cluster of unclassified PMVs but also showed high similarities to other *Miniopterus* PMVs of the genus Jeilongvirus. The phylogenetic reconstruction is based on a 473 nt sequence fragment of the L gene. In general, this highly conserved area coding for the RdRP gene is suitable for phylogenetic analyses, though a reliable classification of the novel strains can only be performed with the complete sequence of the L gene according to ICTV classification criteria [1]. The massive diversity of the family Paramyxoviridae results in difficulties regarding a proper classification system since the establishment of the order Mononegavirales in 1991. Until today, this order was emended 10 times; the last ICTV update was released in 2019 [16,17]. Still, the general demarcation criterion of RdRP nucleotide sequences seems to be insufficient for proper classification. Other criteria, such as host range and host-specific receptor binding proteins (RBPs), biological context, pairwise analysis of sequence complementarity (PASC) for all ORFs, and the size of the P gene, are suggested to classify PMVs properly [18]. In return, this would increase the complexity of PMV classification significantly. Recent studies proposed Shaanvirus as a novel genus within the Orthoparamyxovirinae subfamily [19,20]. The concerned PMVs were first isolated in Korea from *Miniopterus schreibersii* bats and characterized using the above-mentioned criteria. In our analyses, we included Shaanvirus sequences and found a high similarity to the three novel PMV strains from Sri Lanka. Deeper sequence analyses of the whole P gene and genome sequences will be necessary to allow for reliable statements and species proposals. 

For instance, the investigation of the RBP Hemagglutinin (H), Hemagglutinin/Neuraminidase (HN) or attachment glycoprotein (G) are important motifs for the receptor-specific entry to the host cells and can give information about the zoonotic potential of the virus.

In general, bats from the genus Miniopterus were found to host a number of PMVs all over the world. In our study, we investigated bats from Wavul Galge, a sympatric cave for bats from the genera Miniopterus, Rhinolophus, Hipposideros and Rousettus. Although all of these bat genera are known to host PMVs, we detected the novel PMV strains only in the *M. fuliginosus* species [3]. This may be an indication of a host specificity of the novel PMVs to *M. fuliginosus*, which in return may be a result of the presumed co-speciation of the virus and the bat species [5]. Our comparative sequence analyses support this assumption, showing high similarities of PMVs that were found in bats of the genus Miniopterus. However, the number of collected urine samples from the other bat species in the cave is too low to make a final statement about host specificity. 

Paramyxoviruses such as Hendra and Nipah viruses show a seasonal shedding pattern, which is presumably influenced by environmental factors [21]. The presented results suggest a seasonality of the novel PMVs as well. We found PMVs only in July 2018; at that point of the year, *M. fuliginosus* used the Wavul Galge cave as pre-maternity location and was overrepresented [22,23,24]. Consequently, we collected a significantly high number of urine samples from *M. fuliginosus* during this sampling, resulting in a 9.8% positive rate for the novel PMV strains in *M. fuliginosus* bats. At other sampling times, we could not detect PMVs in any of the bat genera inhabiting the cave, though the number of collected urine samples was comparably low at these sampling points. It may be possible that other bat species in the cave carry PMVs as well but show different shedding patterns. Therefore, further samplings of all bat species at different time points over the year could help to understand the seasonality of the novel PMV strains and investigate the presence of PMVs in other bat species inhabiting the cave. Further investigation of all collected samples with different virus detection methods can be used to obtain a comprehensive picture of the prevalence of PMV. Successful virus isolation and NGS analyses may reveal more viral sequence information and allow for further classification of the novel PMVs. 

## 4. Materials and Methods

The investigative research on Sri Lankan bats was approved by the local governmental authority (Department of Wildlife Conservation, Sri Lanka, permit No. WL/3/2/05/18, issued 10 January 2018) and conducted in accordance with relevant guidelines and regulations. Bat sampling from cave-dwelling bats roosting in the Wavul Galge cave (Koslanda, Sri Lanka) was performed in March and July 2018 and January 2019 [9,12]. During the sampling procedures, different sample types were taken if available (feces or rectal swabs, oral swabs, blood, urine swabs). The urine swab samples were taken directly from the bat if available and snap-frozen in liquid nitrogen before storage at −80 °C. For further processing, 500 µL of sterile PBS were added to the urine swab and mixed by vortexing. RNA was extracted using the Viral RNA Mini Kit (QIAGEN, Hilden, Germany). The paramyxovirus screening was performed with a semi-nested PCR assay, targeting an overall 662 nt sequence (first round) on the highly conserved RNA polymerase L gene of paramyxoviruses [25]. The original protocol was slightly adapted, using the AgPath-ID™ One-Step RT-PCR Kit (Applied Biosystems, Waltham, MA, USA) for the first round of the nested PCR assay and 300 nmol each of forward and reverse primer. A total of 2 µL of extracted RNA were used in a final reaction volume of 25 µL. The PCR mixture was incubated at 45 °C for 15 min for the reverse transcription step, followed by an initial denaturation step at 95 °C for 10 min. Forty cycles of PCR were performed at 95 °C for 15 s, 50 °C for 30 s and 72 °C for 30 s, followed by a final incubation step at 72 °C for 6 min. 

For the second round of the semi-nested PCR assay a Platinum Taq DNA Polymerase Kit (Invitrogen, Carlsbad, CA, USA) was used. For each reaction, 300 nmol of forward and reverse primer, 2.5 mM MgCl2, 250 µM dNTPs, 1 × Platinum Taq Buffer and 1.25 U of Platinum Taq DNA polymerase were used. Water was added to a final volume of 23 µL and 2 µL of the first-round PCR product were added. Thermal cycling of the second PCR round was initialized with a denaturation at 95 °C for 2 min, followed by 45 cycles at 95 °C for 15 s, 50 °C for 30 s, 72 °C for 30 s and a final incubation at 72 °C for 6 min. 

Products of both PCR rounds were run and analyzed simultaneously on a 1.5% agarose gel containing DNA Stain G (SERVA, Heidelberg, Germany). Samples showing a distinct band in the gel analysis were purified by using MSB Spin PCRapace Kit (Invitrogen, Carlsbad, CA, USA) and sequenced with a BigDye Terminator Cycle Sequencing Kit on a 3500 Dx Genetic Analyzer (Applied Biosystems, Waltham, MA, USA), using the corresponding forward and reverse primers for each strand. 

Sanger sequences were analyzed and checked for quality by using Geneious Prime software, low-quality bases at the end of each sequence were trimmed before further processing. 

A de novo assembly of all sanger sequences was calculated using Velvet assembler [26]. The resulting sequences are available online at GenBank and are named batPMV/MinFul/SL/2018_1 (Accession number OM777173), batPMV/MinFul/SL/2018_2 (Accession number OM777172) and batPMV/MinFul/SL/2018_3 (Accession number OM777171). 

A nucleotide alignment of 473 nt was calculated by using MAFFT algorithm v7.450 [27]. The alignment contained the three novel strains from Sri Lanka as well as different PMV representative strains from different Orthoparamyxovirinae genera downloaded from the NCBI database. A phylogenetic tree was calculated by using MrBayes version 3.2.6 [28]. The model GTR with gamma-distributed rate variation was selected for these calculations; parameters were set as follows: number of runs: four; number of generations: 1,000,000; subsampling frequency: 100; and burn in: 10%. The reference strain avian pneumovirus LAH A (NC_039231) was selected as outgroup for the calculations. The phylogenetic tree was visualized with Geneious Prime software. 

## 5. Conclusions

With this study, we detected PMVs for the first time in *M. fuliginosus* and for the first time in Sri Lanka. Sequence analysis revealed the presence of different strains with high similarity to other unclassified PMVs. The results indicate a high host-specificity of the novel strains batPMV/MinFul/SL/2018_1–3 to the bat species *M. fuliginosus*; we did not detect the novel strains in other bat species inhabiting the cave. Studying the sympatric bat species in Wavul Galge, one of the largest caves in Sri Lanka, can give important insights to virus–host interaction, host specificity and the seasonal shedding of viruses. Therefore, we suggest a long-term monitoring of the bats and their viruses in the cave, whereby a respectful interaction is important to protect their natural habitat.

## Figures and Tables

**Table 1:** Overview of the urine samples (PMV-positive/total of samples) collected from different bat genera at three sampling points.

Genus	March 2018	June 2018	January 2019	Total Urine Samples
**Miniopterus**	0/0	10/102	0/11	**10/113**
**Rousettus**	0/2	0/2	0/6	**0/10**
**Hipposideros**	0/2	0/0	0/6	**0/8**
**Rhinolophus**	0/6	0/0	0/6	**0/12**

## Data Availability

The data presented in this study are openly available in GenBank (https://www.ncbi.nlm.nih.gov/genbank/, accessed on 1 March 2022, Accession number OM777171–OM777173).
